# Supplementary material for: Improved Characterization of Circulating Tumor Cells and Cancer-Associated Fibroblasts in One-Tube Assay in Breast Cancer Patients Using Imaging Flow Cytometry
Source: Cancers (Basel). 2023 Aug 18;15(16):4169. doi: 10.3390/cancers15164169 (PMC10453498; doi:10.3390/cancers15164169)
Supplement: Supplementary file 1 [file cancers-15-04169-s001.zip › Supplementary Figure S1.pdf]

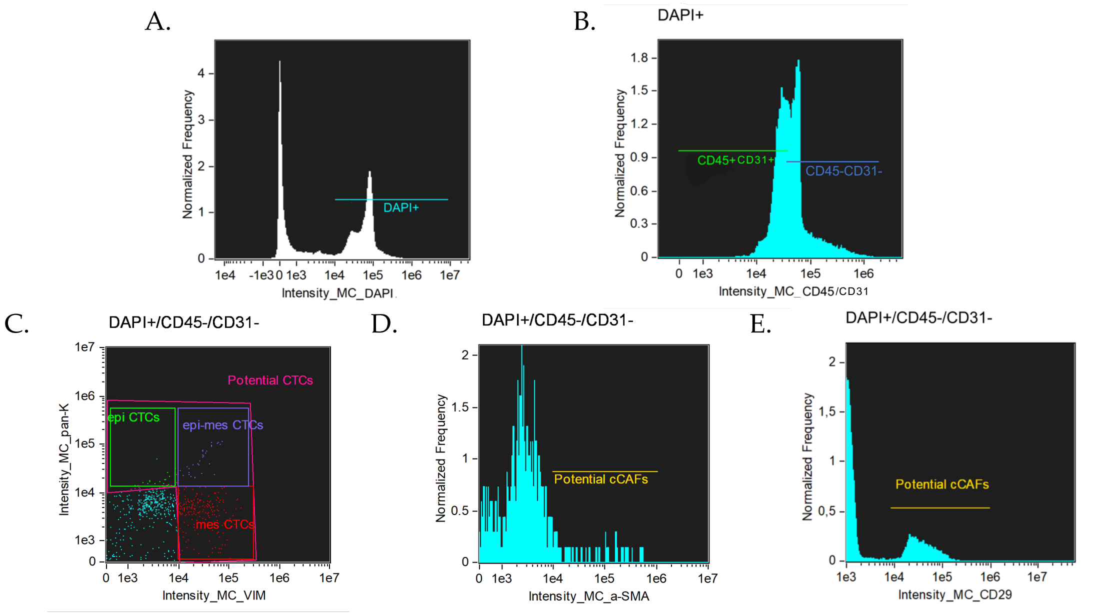

Fig. S1 Gating strategy. A. All DAPI+ cells were counted, **B.** DAPI+/CD45-CD31- cells were collected. **C.** To gate potential CTCs, fluorescence intensity of K and V were visualized in a 2D dot plot. To gate cCAFs, fluorescence intensity of **D.** α-SMA and **E.** CD29 was visualized in histograms.
