# Supplementary material for: Improved Characterization of Circulating Tumor Cells and Cancer-Associated Fibroblasts in One-Tube Assay in Breast Cancer Patients Using Imaging Flow Cytometry
Source: Cancers (Basel). 2023 Aug 18;15(16):4169. doi: 10.3390/cancers15164169 (PMC10453498; doi:10.3390/cancers15164169)
Supplement: Supplementary file 1 [file cancers-15-04169-s001.zip › Supplementary Figure S2.pdf]

LumA

T-47D

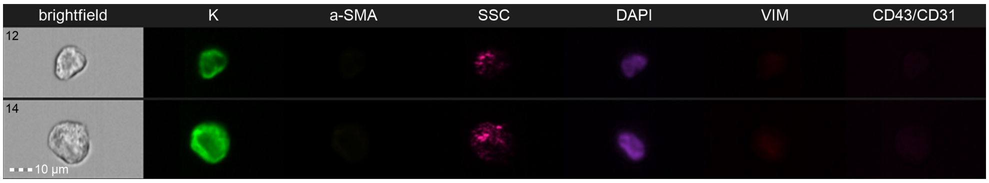

MCF7

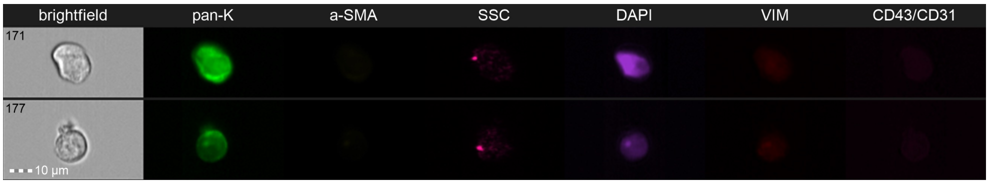

LumB

BT-474

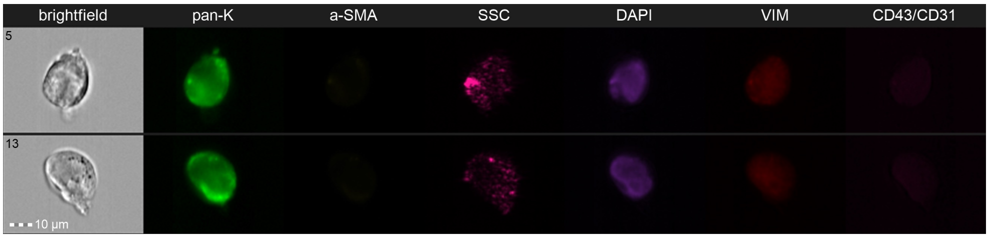

MDA-MB-361

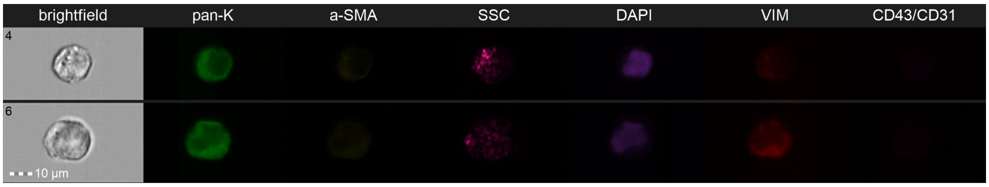

HER2+

SKBR3

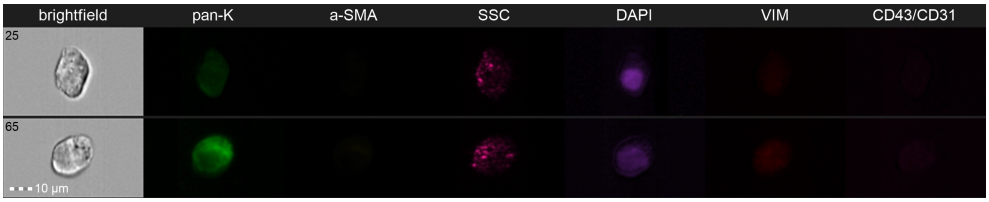

TNBC

HCC1806

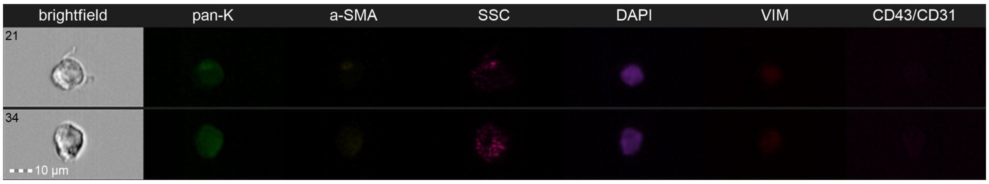

MDA-MB-231

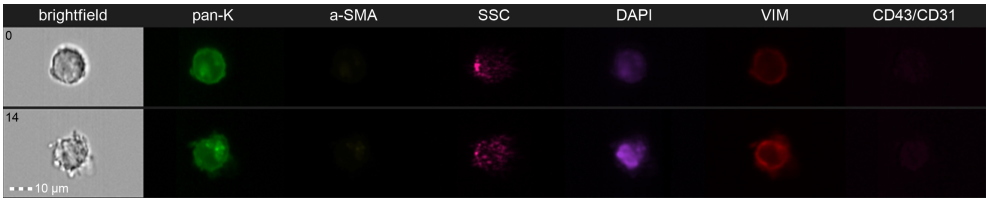

Fig. S2 Representative pictures of different breast cancer (BC) cell lines corresponding different BC molecular subtypes envisioned by imaging flow cytometry.
