# Supplementary material for: Improved Characterization of Circulating Tumor Cells and Cancer-Associated Fibroblasts in One-Tube Assay in Breast Cancer Patients Using Imaging Flow Cytometry
Source: Cancers (Basel). 2023 Aug 18;15(16):4169. doi: 10.3390/cancers15164169 (PMC10453498; doi:10.3390/cancers15164169)
Supplement: Supplementary file 1 [file cancers-15-04169-s001.zip › Supplementary Table.pdf]

Supplementary Table S1
Antibodies dilution and acquisition parameters used for ImFC.

Antibody	Company	Cat. nr.	Fluorophor	Dilution	Laser	Laser power	Channel
BF							1+9
pan-keratin (AE1/AE3)	Thermo Fisher Scientific	53-9003-82	Alexa 488	1:2500	488	100mW	2
Pan-keratin (C11)	Thermo Fisher Scientific	MA5-18156	Alexa 488	1:2500	488	100mW	2
a-SMA (1A4)	R&Dsystems	IC1420P	PE	1:50	488	100mW	3
SSC					785		6
DAPI	BD Biosciences	564907	DAPI	0,04ug/ml	405	100mW	7
CD29 (TS2/16)	Thermo Fisher Scientific	63-0299-42	SuperBright600	1:1000	405	100mW	10
Vimentin (D21H3)	Cell Signaling	9856	Alexa 647	1:100	647	150mW	11
CD45 (REA747)	Miltenyi Biotec	130-110-635	APC-Vio770	1:50	647	150mW	12
CD31 (WM59)	BioLegend	303120	APC-Cy7	1:10	647	150mW	12

Supplemental Table S2
Correlations of CTC phenotypes with cCAFs.

		epi CTCs	epi-mes CTCs	mes CTCs	neg CTCs	cCAFs
**epi CTCs**	R^2	1	-0.015	**0.259**	**0.772**	**0.165**
	p-values		0.813	**<0.001**	**<0.001**	**0.017**
	n	210	210	**210**	**210**	**210**
**epi-mes CTCs**	R^2		1	0.086	0.105	0.086
	p-values			0.215	0.128	0.214
	n		210	210	210	210
**mes CTCs**	R^2			1	**0.314**	0.097
	p-values				**<0.001**	0.163
	n			210	**210**	210
**neg CTCs**	R^2				1	**0.250**
	p-values					**<0.001**
	n				210	**210**
**cCAFs**	R^2					1
	p-values					
	n					210

Supplementary Table S3

Comparison of exclusive CTC phenotypes among clinico-pathological features and response to treatment of patients with breast cancer. Chi squared test was performed. Due to missing data not all numbers sum up to 210.

		**Exclusive CTC phenotypes**					
Variable	total n	epi	mes	epi-mes	neg	heterogeneous	p-value
**Age**							
<50	16	2 (18.2%)	2 (12.5%)	2 (66.75)	0 (0%)	10 (43.5%)	**0.02**
>>50	42	9 (81.8%)	14 (87.5%)	1 (33.3%)	5 (100%)	13 (56.5%)	
**cT stage**							
cT1-2	27	5 (62.5%)	8 (72.7%)	2 (100%)	2 (50%)	10 (66.7%)	0.8
cT3-4	13	3 (37.5%)	3 (27.3%)	0 (0%)	2 (50%)	5 (33.3%)	
**cN stage**							
0	15	5 (62.5%)	4 (40%)	2 (100%)	0 (0%)	4 (25%)	0.11
1	25	3 (37.5%)	6 (60%)	0 (0%)	4 (100%)	12 (75%)	
**M stage**							
0	46	7 (63.6%)	12 (75%)	3 (100%)	5 (100%)	19 (82.6%)	0.05
1	12	4 (36.4%)	4 (25%)	0 (0%)	0 (0%)	4 (15.4%)	
**Grading**							
1	3	1 (11.1%)	1 (8.3%)	0 (0%)	0 (0%)	1 (5%)	0.46
2	26	3 (33.3%)	7 (58.3%)	3 (100%)	4 (100%)	9 (45 %)	
3	19	5 (55.6%)	4 (33.3%)	0 (0%)	0 (0%)	10 (50%)	
**Molecular type**							
Luminal A	9	1 (10%)	4 (25%)	1 (33.3%)	1 (25%)	2 (8.7%)	0.1
Luminal B HER2-	14	1 (10%)	4 (25%)	2 (66.7%)	0 (0%)	7 (30.4%)	
Luminal B HER2+	13	2 (20%)	4 (25%)	0 (0%)	3 (75%)	4 (17.4%)	
Non luminal HER2+	3	0 (0%)	1 (6.3%)	0 (0%)	0 (0%)	2 (8.7%)	
Triple Negative	17	6 (60%)	3 (18.8%)	0 (0%)	0 (0%)	8 (34.8%)	
**Response to treatment**							
stable disease	6	0 (0%)	2 (25%)	1 (100%)	2 (50%)	1 (8.3%)	0.45
complete response	9	2 (50%)	1 (12.5%)	0 (0%)	1 (25%)	5 (41.7%)	
partial response	13	2 (50%)	5 (62.5%)	0 (0%)	1 (25%)	5 (41.7%)	
progressive disease	1	0 (0%)	0 (0%)	0 (0%)	0 (0%)	1 (8.3%)	

Supplementary Table S4

Distribution of dominant CTC phenotypes among clinico-pathological features and response to treatment of patients with breast cancer. Chi squared test was performed. Due to missing data not all numbers sum up to 210.

		**Dominant CTC phenotypes**					
Variable	total n	epi	mes	epi-mes	neg	heterogenous	p-value
**Age**							
<50	16	6 (27.3%)	3 (17.6%)	4 (66.7%)	2 (20%)	1 (33.3%)	0.08
>>50	42	16 (73.7%)	14 (82.4%)	2 (33.3%)	8 (80%)	2 (66.7%)	
**cT stage**							
cT1-2	27	10 (71.4%)	8 (66.7%)	3 (100%)	4 (50%)	2 (66.7%)	0.65
cT3-4	13	4 (28.6%)	4 (33.3%)	0 (0%)	4 (50%)	1 (33.3%)	
**cN stage**							
0	15	6 (40%)	4 (36.4%)	2 (66.7%)	2 (25%)	1 (33.3%)	0.88
1	25	9 (60%)	7 (63.6%)	1 (33,3%)	6 (75%)	2 (66.7%)	
**M stage**							
0	46	16 (72.7%)	13 (76.5%)	4 (66.7%)	10 (100%)	3 (100%)	**0.03**
1	12	6 (23.1%)	4 (23.5%)	2 (33.3%)	0 (0%)	0 (0%)	
**Grading**							
1	3	2 (11.1%)	1 (7.7%)	0 (0%)	0 (0%)	0 (0%)	0.84
2	26	8 (44.4%)	7 (53.8%)	4 (80%)	6 (66.7%)	1 (33.3%)	
3	19	8 (44.4%)	5 (38.5%)	1 (20%)	3 (33.3%)	2 (66.7%)	
**Molecular type**							
Luminal A	9	2 (9.5%)	4 (23.5%)	1 (16.7%)	2 (22.2%)	0 (0%)	0.17
Luminal B HER2-	14	4 (19%)	4 (23.5%)	3 (50%)	1 (11.1%)	2 (66.7%)	
Luminal B HER2+	13	3 (14.3%)	4 (23.5%)	2 (33.3%)	4 (44.4%)	0 (0%)	
Non luminal HER2+	3	1 (4.8%)	1 (5.9%)	0 (0%)	1 (11.1%)	0 (0%)	
Triple Negative	17	11 (52.4%)	4 (23.5%)	0 (0%)	1 (11.1%)	1 (33.3%)	
**Response to treatment**							
stable disease	6	0 (0%)	2 (25%)	2 (100%)	2 (28.6%)	0 (0%)	0.19
complete response	9	4 (44.4%)	1 (12.5%)	0 (0%)	3 (42.9%)	1 (33.3%)	
partial response	13	4 (44.4%)	5 (62.5%)	0 (0%)	2 (28.6%)	2 (66.7%)	
progressive disease	1	1 (11.1%)	0 (0%)	0 (0%)	0 (0%)	0 (0%)	
