# Supplementary figures and images for: Improved Characterization of Circulating Tumor Cells and Cancer-Associated Fibroblasts in One-Tube Assay in Breast Cancer Patients Using Imaging Flow Cytometry
Source: Cancers (Basel). 2023 Aug 18;15(16):4169. doi: 10.3390/cancers15164169 (PMC10453498; doi:10.3390/cancers15164169)

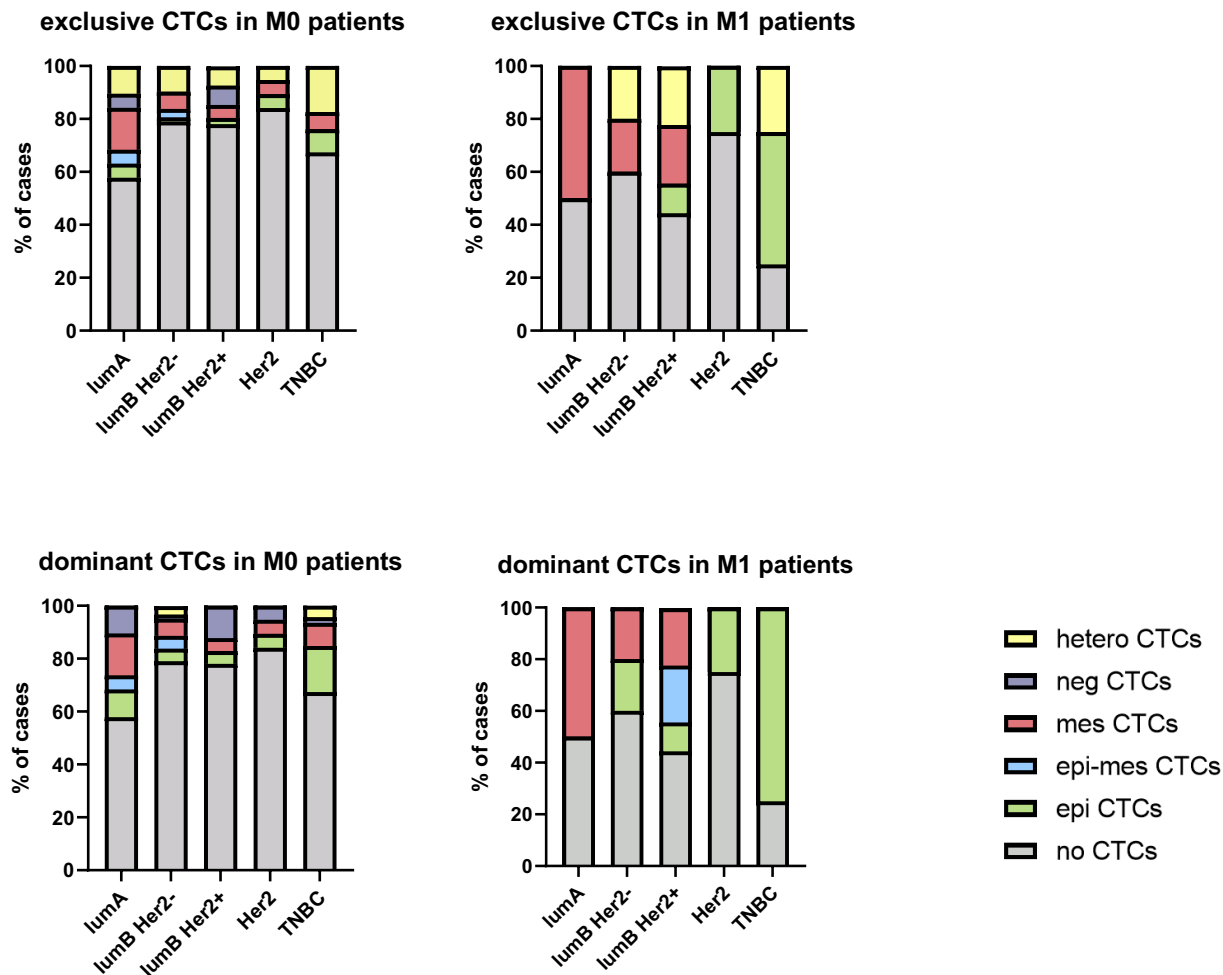

**Fig. S3** Distribution of exclusive and dominant CTCs phenotypes in M0 and M1 breast cancer patients.

Supplement: Supplementary file 1 [file cancers-15-04169-s001.zip › Supplementary Figure S3.pdf]
